# The First Peopling of South America: New Evidence from Y-Chromosome Haplogroup Q

**DOI:** 10.1371/journal.pone.0071390

**Published:** 2013-08-21

**Authors:** Vincenza Battaglia, Viola Grugni, Ugo Alessandro Perego, Norman Angerhofer, J. Edgar Gomez-Palmieri, Scott Ray Woodward, Alessandro Achilli, Natalie Myres, Antonio Torroni, Ornella Semino

**Affiliations:** 1 Dipartimento di Biologia e Biotecnologie “Lazzaro Spallanzani”, Università di Pavia, Pavia, Italy; 2 Sorenson Molecular Genealogy Foundation, Salt Lake City, Utah, United States of America; 3 AncestryDNA, Provo, Utah, United States of America; 4 Dipartimento di Chimica, Biologia e Biotecnologie, Università di Perugia, Perugia, Italy; 5 Centro Interdipartimentale “Studi di Genere”, Università di Pavia, Pavia, Italy; University of Utah, United States of America

## Abstract

Recent progress in the phylogenetic resolution of the Y-chromosome phylogeny permits the male demographic dynamics and migratory events that occurred in Central and Southern America after the initial human spread into the Americas to be investigated at the regional level. To delve further into this issue, we examined more than 400 Native American Y chromosomes (collected in the region ranging from Mexico to South America) belonging to haplogroup Q – virtually the only branch of the Y phylogeny observed in modern-day Amerindians of Central and South America – together with 27 from Mongolia and Kamchatka. Two main founding lineages, Q1a3a1a-M3 and Q1a3a1-L54(xM3), were detected along with novel sub-clades of younger age and more restricted geographic distributions. The first was also observed in Far East Asia while no Q1a3a1-L54(xM3) Y chromosome was found in Asia except the southern Siberian-specific sub-clade Q1a3a1c-L330. Our data not only confirm a southern Siberian origin of ancestral populations that gave rise to Paleo-Indians and the differentiation of both Native American Q founding lineages in Beringia, but support their concomitant arrival in Mesoamerica, where Mexico acted as recipient for the first wave of migration, followed by a rapid southward migration, along the Pacific coast, into the Andean region. Although Q1a3a1a-M3 and Q1a3a1-L54(xM3) display overlapping general distributions, they show different patterns of evolution in the Mexican plateau and the Andean area, which can be explained by local differentiations due to demographic events triggered by the introduction of agriculture and associated with the flourishing of the Great Empires.

## Introduction

Three main events contributed to the genetic structure of modern American populations: the entry into the Americas of Beringian and Asian groups which gave rise to the First Americans, the internal movements determined by the demographic expansions ascribable to the agricultural advances and/or the flourishing of the Great Native American Empires and, finally, the European and African post-Colombian gene-flow. Due to the high rate of admixture between autochthonous people and newcomers from the Old World [1], most of the present genetic information on Native Americans derives from studies of **uniparental markers** [2]. Recently, clues on the origin and spread of the First Americans have been provided by analyses at the level of entire **mitochondrial genomes** [3]–[12], which revealed a higher level of molecular diversity in the Americas than previously reported.

Several studies have focused on the **Y chromosome** as an independent source of genetic information, but the identification of the male founding lineages has been particularly challenging because of the drastic reduction among modern American populations of autochthonous Y-chromosomes following the male post-Colombian colonization and the high rate (about 17%) of Old World male-mediated admixture [13]. Nevertheless, two founding lineages of Asian ancestry, designated C and Q, were described early on [13]–[20] and, on average, they account for about 6% and 75% of Native American Y chromosomes, respectively. According to the multimodal distribution of these haplogroups and the different geographic patterns of their sub-clades, their entry into the Americas is likely to have occurred at different times, with haplogroup Q (observed all over the double continent) arriving prior to haplogroup C (limited to North America). During the last two decades, the resolution of these haplogroups has not undergone substantial improvements, with the vast majority of Native American Y chromosomes still falling into one of the two main lineages, Q1a3a1a-M3 [14] and C3b-P39 [13].

As attested by recent mitochondrial DNA (mtDNA) studies, only the dissection of the most common Y-chromosome founding haplogroups, together with major sampling efforts, can permit the identification of geographically-restricted lineages able to provide information not only on the migration routes but also on the demographic dynamics of the male component associated with the population expansions that occurred at different times and locations in the American continent. With this aim, more than 400 Native American unrelated male subjects, harboring Y chromosomes belonging to haplogroup Q were identified among more than 2,000 subjects of paternal Native American ancestry from a broad geographic area, ranging from Mexico to South America. These subjects have been examined for 23 haplogroup Q-specific SNPs and 37 Y-STR markers. In addition, in the attempt to identify possible genetic sources for Paleo-Indian Y chromosomes, the results obtained were analyzed in comparison with those of different populations, geographically or culturally related to the proposed ancestral Asian source areas, some of them already published, others (the Koryaks from Kamchatka and the Mongols) analyzed in the present study.

### The first peopling of America: state of the art

Since 1590, when Friar José de Acosta first argued that the indigenous inhabitants of the Americas likely came from Asian populations [21], the events that characterized this process of colonization of the Americas, the last major landmass to experience human dispersal, have been a matter of debate. Scholars of different fields, including archaeologists, anthropologists, linguists and geneticists seek to answer questions concerning the number of migrations, the homelands of the peoples involved in the process and the routes they followed, as well as the timing of these events.

The traditional model of colonization, first advanced by Hrdlička [22] and subsequently modified in order to incorporate new chronological and cultural data [23], [24], assumed a single rapid migration (containing all founding lineages) of Asian ancestors across the Bering land bridge around 16.5 thousand years ago (kya – calendar years BP) [25], the earliest period for which there are significant archaeological findings on both side of Beringia. The basis of this model, called the “blitzkrieg” model, was the identification of the Clovis culture, the oldest (∼13 kya) and most widely distributed archaeological tradition in North America [26]. Later, Greenberg et al. [27] combining linguistic, archaeological and biological evidence, proposed the “three-wave” model according to which the three proposed Native American linguistic groups (Amerind, Na-Dene, and Eskimo-Aleut) entered the Americas subsequently to the Last Glacial Maximum (LGM, ∼20 kya), in three chronologically separate waves of migrations from different Asian regions. Afterwards, different models were advanced to explain observations based on new genetic data: some were in agreement with the three waves of migration, [28], [29] whereas others proposed a single early entry into the Americas [30], [31] or two waves of migration, the first from Central Asia (Altai-Sayan and/or mid-lower-Amur) region prior to LGM, and the second, after the LGM, from Amur-Mongolia-Manchuria [32]. Recently, after extensive consideration of the linguistic classification and the genetic differentiation of the three Native American family groups [2], [33], [34], the latest genetic screening of the nuclear genome supports a model with at least “three migration-waves”, with a major contribution from the first arrival, accounting for 100% of the Amerindian ancestry in most Native American populations and with decreasing contributions from the subsequent two migrations, which mainly affected Na-Dene and Eskimo-Aleut groups [35]. In addition, the presence and high frequency of alleles and haplogroups unique to Native Americans (such as the autosomal 9-repeat at microsatellite locus D9S1120 [36], [37], the Y-chromosome haplogroup Q1a3a-M3 [20], [38], [39] and the pan-American mtDNA haplogroups A2, B2, C1b, C1c, C1d, D1, and D4h3a [3]–[7]) support the scenario that Beringian/Eastern Siberian glacial refuges existed and played a major role in shaping the ancestral gene pool of Native Americans. Here, founder lineages differentiated from their Asian sister-clades by genetic drift prior to entering North America. Then, they followed a swift migration southward throughout the rest of the double continent [3], [7], [37]. Some have proposed a more complex model, in which the initial differentiation from Asian populations ended with a moderate bottleneck in Beringia during the LGM [5]. Thus, a subsequent remarkable population expansion occurred with rapid settlement along a coastal route, the only one available at that time (from 18 to 15 kya), shortly before the development and spread of the Clovis culture [40]. Recently, through the analysis of the distribution of two rare Native American mtDNA haplogroups, a second major path of migration was identified along the interior ice-free corridor between the Laurentide and Cordilleran ice sheets, more or less concomitantly (17–15 kya) to the principal migration along the Pacific coast [6], [41].

Regarding South America, taking into account the archaeological evidence of pre-Clovis cultures, such as Monte Verde in Chile (∼15 kya) [42] and the Oregon cave (∼14 kya) [43], it is likely that people arrived in the Southern Hemisphere no later than 15 to 14 kya [42], [44]. Nevertheless, archaeological evidence (tools and skills for harvesting resources from the sea, such as fish, shellfish, and sea mammals) of sufficient antiquity to account for the first arrivals in southern North America is limited to the Paleo-coastal sites in the California Channel Islands, dated 12–11 kya [45]. Simulation models and archaeological records [42], [46] suggest the initial settlement along the coasts was followed by colonization along rivers while recent analyses of mitochondrial genomes from the Southern Cone of South America point strongly to a trans-Andean colonization of the continental interior after the southward coastal migration [9]–[11].

The first inhabitants of the American continent were hunter-gatherers and fishers, as witnessed by archaeological records [47]. Climatic changes and subsequent environmental changes pushed humans and animals towards new areas with favorable conditions to which, in spite of highly differing environments, they adapted rapidly thanks to the relative homogeneity of available prey along the whole American continent. As already hypothesized for Asia and Europe, there was most probably demographic growth just before the invention of agriculture, which drove humans to develop new technologies to meet the growing need for food. However, this process was relatively slow compared to Eurasia, due to differing and unique environments requiring specific technologies that could not be easily exported into neighboring territories. Ancient cultivations (more than 10–8 kya) [48] were identified only in present-day Mexican territories and in the western side of South America, the locations of the largest American Empires such as the Maya – the longest-lasting civilization in Mesoamerica (∼3,500–500 ya), and the Aztecs and Incas (∼1,000–500 ya). However, hunting and fishing were maintained elsewhere, giving rise to highly structured societies. The Aztecs, who came to the South of present day Mexico from dry uplands of the North, achieved hegemony when the Mayan Empire collapsed. At the same time, in South America, the Incas were able to extend their control 2,500 miles along the Andes, ruling up to 20 different language areas, until the arrival of the Spanish invaders. Investigating the gene pool of these regions, which cover today at least a portion of Ecuador, Peru, Bolivia, Chile and Argentina, along with Mesoamerica, could provide important insights into the migration flow and expansion which, from Beringia, eventually arrived to Central and South America.

## Results

**Figure 1:**
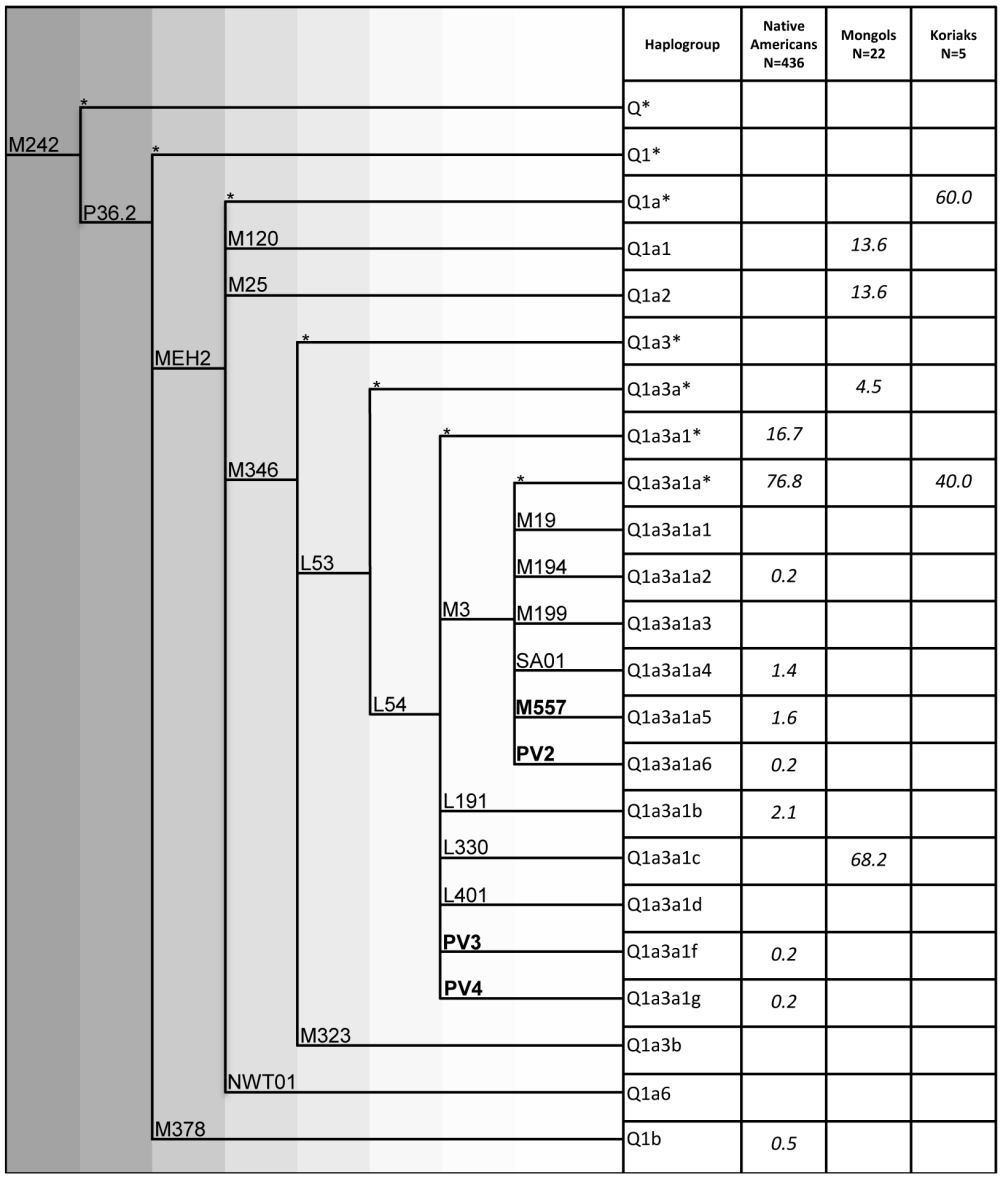
Phylogenetic tree of Y-chromosome haplogroup Q. Haplogroup nomenclature and frequencies (*%*) of haplogroup branches in the analyzed populations (new markers are in bold). The haplogroup labeling is in agreement with the YCC [85] conventions and recent updates [49], [51]. *Paragroups: Y chromosomes not defined by any phylogenetic downstream-reported and -examined mutation.

As reported in Table 1, 463 Y chromosomes belonging to haplogroup Q (M242-positive) were identified in this survey. All of them were analyzed hierarchically for signature markers of all haplogroup Q sub-lineages and for new informative variants identified while typing. The analyzed markers and the relative results are shown in Figure 1, which also illustrates the phylogenetic position of the four new lineages.

**Table 1:** Observed Frequencies of Haplogroup Q and of its Sub-Lineages.

Macro-area	Country or Region *(N)*	Q (N)	Q1a- MEH2	Q1a1- M120	Q1a2- M25	Q1a3a- L53*	Q1a3a1- L54*	Q1a3a1a- M3*	Q1a3a1a2- M194	Q1a3a1a4- SA01	Q1a3a1a5- M557^a^	Q1a3a1a6- PV2^a^	Q1a3a1b- L191	Q1a3a1c- L330	Q1a3a1f- PV3^a^	Q1a3a1g- PV4^a^	Q1b- M378
Asia	Mongolia *(1,145)*	**22**		*3 (13.6)*	*3 (13.6)*	*1 (4.5)*								*15 (68.2)*			
	Kamchatka *(34)*	**5**	*3 (60.0)*					*2 (40.0*)									
Mexico	Mexico *(485)*	**181**					*48 (26.5)*	*123 (68.0)*					*8 (4.4)*		*1 (0.6)*	*1 (0.6)*	
Central America	C American Isthmus^b^ *(14)*	**3**						*3 (100.0)*									
	Nicaragua *(10)*	**2**					*2 (100.0)*										
	Costa Rica *(35)*	**1**						*1 (100.0)*									
	Panama *(242)*	**37**					*2 (5.4)*	*34 (91.9)*									*1 (2.7)*
Andean Regions	NS America^b^ *(54)*	**9**					*2 (22.2)*	*6 (66.7)*									*1 (11.1)*
	Peru *(397)*	**159**					*17 (10.7)*	*126 (79.2)*	*1 (0.6)*	*6 (3.8)*	*7 (4.4)*	*1 (0.6)*	*1 (0.6)*				
	Bolivia *(22)*	**9**					*1 (11.1)*	*8 (88.9)*									
	Chile *(206)*	**20**						*20 (100.0)*									
South East America	Brazil *(485)*	**7**						*7 (100.0)*									
	SS America^b^ *(18)*	**7**					*1 (14.3)*	*6 (85.7)*									
	Uruguay *(37)*	**1**						*1 (100.0)*									
Caribbean Region	The Caribbeans^b^ *(20)*	**–**															
	Total *(3,204)*	**463**															

a Marker described here for the first time.

b Grouping due to sample size <10 in single populations. Central American Isthmus: El Salvador, Guatemala and Honduras; Northern South America: Colombia, Ecuador, Venezuela; Southern South America: Argentina and Paraguay; The Caribbeans: Barbados, Bermuda, Cuba, Dominican Rep., French Guiana, Grenada, Haiti, Jamaica, Martinique.

The following mutations were surveyed in all potential carrier samples but they were not observed: NTW01, M323, L401 and M19. M199 was only typed in a subset of samples.

In parentheses, % frequencies.

### New lineages

In the most updated phylogeny of haplogroup Q shown in Figure 1 [49], the four new markers define two new M3 sister lineages (PV3 and PV4) and two M3 sub-lineages (M557 and PV2). M557 was identified while typing by sequencing the M194 marker and consists of a TAC deletion in a TACTAC repeat of its amplicon; PV2, PV3, and PV4 are all T to C transitions discovered while sequencing the M242, L330 and L54 markers, respectively (for details see Table S1). The haplogroup Q Y-chromosome sequenced by Wei and colleagues [50] does not harbor any of these new markers.

### Haplogroup distribution

The uneven distribution of haplogroup Q1a3a1-L54 in Native American and Asian samples recently described by [49] was confirmed. This clade harbors virtually all (99.5%) the Native American Y chromosomes. However, when taking into account also the Altaian samples of Dulik et al. [51], it accounts for only 51% of Q Y chromosomes from Asian populations living in the regions that have been proposed as sources and pathways of Native American migrations (Figure 1, Table 1 and Dulik et al. [51]). In addition, with the only exception of two Koryak samples that were M3-positive, none of the observed lineages are shared between Native Americans and Asians, 80% of the first falling into the Q1a3a1a sub-clade characterized by the biallelic marker M3 and 49% of the second harboring the Q1a3a1c sub-clade defined by the L330 marker.

Only two American Y chromosomes did not cluster into the L54 sub-branch. They were both M378-positive, thus belonging to Q1b, a finding never previously reported for Native Americans. Considering that the phylogeography of this infrequent haplogroup is restricted to South West Asia [52]–[55], the most likely interpretation of this outcome is that they represent an arrival from Asia in contemporary history. For this reason the two Y chromosomes were not included in subsequent analyses.

Focusing on the Native American-specific lineages, Q1a3a1a-M3 is prevalent in all considered populations. Q1a3a1-L54(xM3) has mainly been observed in Central America as paragroup Q1a3a1-L54*, while Q1a3a1b-L191 was observed in eight Mexicans and one Peruvian sample, and Q1a3a1f-PV3 and Q1a3a1g-PV4 were found in single Mexican subjects. Q1a3a1d-L401 was not observed in our sample. Regarding M3 sub-branches, Q1a3a1a4-SA01, recently described in 16 subjects from the Andean region [56], and the new Q1a3a1a5-M557, were observed in 1.5% (N = 6) and 1.8% (N = 7) of the Peruvian samples, respectively; lastly, Q1a3a1a2-M194 and Q1a3a1a6-PV2 were found in single Peruvian subjects while the previously described Q1a3a1a1-M19 and Q1a3a1a3-M199 (only typed in a subset of samples) were not observed in this survey.

### STR haplotype distribution among the Native American haplogroup Q sub-lineages

**Figure 2:**
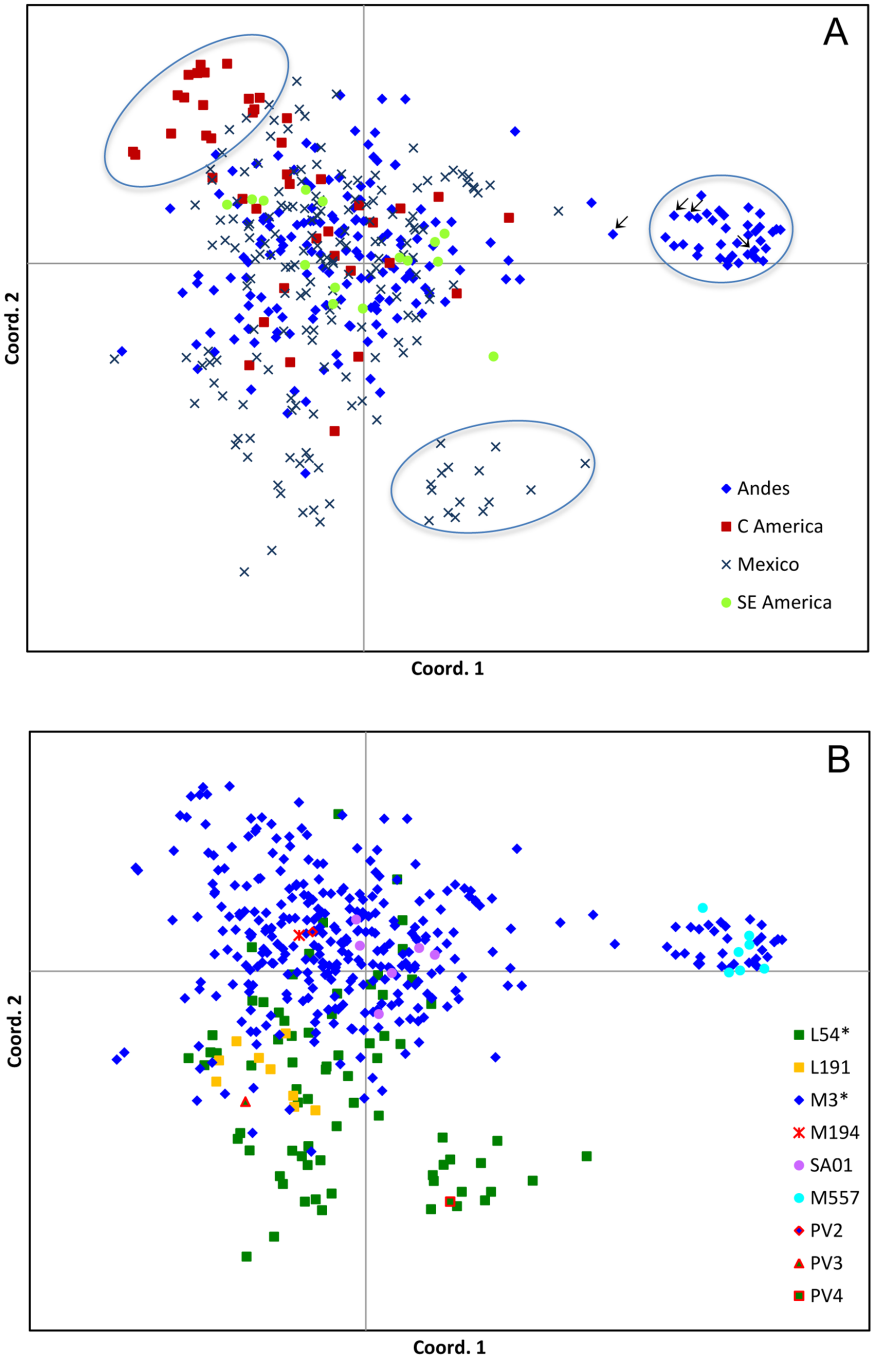
PCoA plots. Analyses were performed on 433 Native American haplogroup Q Y chromosomes and based on pairwise, individual-by-individual genetic distances generated from 33 *loci* Y-STR haplotypes (Table S2) represented in their (A) geographic (Andes: Bolivia, Chile, Colombia, Ecuador, Peru; Central America: Costa Rica, El Salvador, Guatemala, Nicaragua, Panama; South East America: Argentina, Brazil, Paraguay, Uruguay) and (B) sub-haplogroup Q contexts. Ancient DNA (aDNA) matching haplotypes [75] are indicated by arrows. Three separated (Central American, Mexican and Andean) clusters are indicated by ovals.

Figure 2 illustrates the two principal coordinates (PCos) of the 33 STR *loci* variation in the 433 Native American Y-chromosome haplogroup Q samples of the present study (Table S2) in relation to geographic (panel A) and haplogroup (panel B) distributions. The first principal coordinate sharply distinguishes an Andean cluster from the bulk of samples (panel A), while the second coordinate separates the haplotypes related to the M3 background from haplotypes associated with the L54(xM3) context (panel B). Two other well-differentiated groupings stand out from the plots: one harbouring Central American M3 Y chromosomes and the other encompassing a group of Mexican L54(xM3) samples. The location in the plot of the samples carrying the new variants is in agreement with this distribution, with the M557 lineage restricted to the above-mentioned Andean cluster.

High values of haplotype diversity (*h*) were observed for both founding haplogroups L54(xM3)(0.9744) and M3 (0.9915). When geographic areas were considered, L54 displayed high values in Mexico (0.9492) and the Andean (0.9905) (or Peruvian) regions; differently, M3 displayed the highest value in Mexico (0.9897) and a general slightly decreasing north-to-south diversity pattern. In the Far East Asians, M3 showed the lowest value (0.9111) (Table S3 and data not shown).

### Network analyses and time estimates

**Figure 3:**
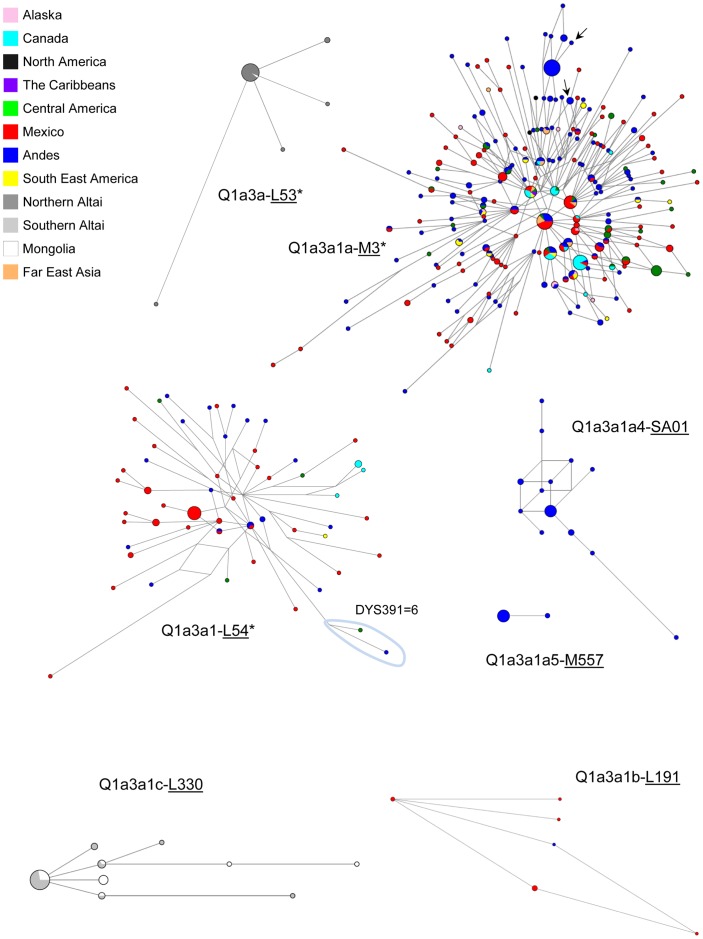
Network of 7 *loci* Y-STR haplotypes belonging to Q sub-haplogroups. Network analysis were performed on Native American [Alaskan, Canadian, North American, Caribbean [49], Central American (from Costa Rica, El Salvador, Guatemala, Nicaragua, Panama), Mexican, Andean (from Bolivia, Chile, Colombia, Ecuador, Peru), South Eastern American (from Argentina, Brazil, Paraguay, Uruguay) (present study)] and Asian [Northern and Southern Altaians [51], Mongolian and Far Eastern Asian (from Kamchatka, Chukotka and Sea of Okhotsk coast) [59], [78]; present study)] samples (Table S4). Light blue loop is referred to the unusual DYS391-6 repeat allele. Arrows indicate the 7 *loci* haplotypes matching aDNA [75]. Within the single network, circles and colored sectors are proportional to the number of subjects, with the smallest circle equal to one Y chromosome; connecting lines are proportional to the number of mutations.

To compare our results with literature data from North America and informative Asian regions, in spite the large number of STR *loci* typed in the present study, network analyses and time estimates of haplogroup Q sub-lineages were performed on the subset of *loci* widely used in evolutionary studies and with well-defined evolutionary mutational rates [57], [58]. The median-joining networks of the seven STR haplotypes within the observed Q sub-haplogroups (Table S4) are shown in Figure 3, whereas the estimated ages are reported in Tables 2 and 3. Interestingly, the two founding lineages display an early concomitant arrival in Mexican and Andean regions, when the above-mentioned clusters, ascribable to later expansions, were disregarded.

**Table 2:** Estimated Ages for Q Sub-Lineages in Native Americans.

Lineage *(N* a *)*	Age ± SE (kya)
Q-total *(431)*	22.2±3.8
Q1a3a1-L54 *(83)*	24.4±3.4^b^
Q1a3a1-L54* *(74)*	23.6±3.8^c^
Q1a3a1a-M3 *(348)*	21.8±4.1
Q1a3a1a-M3* *(334)*	21.0±3.7^d^
Q1a3a1b-L191 *(9)*	3.6±1.6
Q1a3a1-DYS19-13.2 *(6)* e,f	9.4±5.8

SE, Standard Errors.

a excluding samples with duplicated loci and micro-variants (partial repeats).

b including PV4, 24.3±3.4 without PV4.

c including PV4, 23.7±3.7 without PV4.

d including or excluding PV2.

e one sample from this study and five from the YRHD database.

f DYS388 and DYS461 were not genotyped.

**Table 3:** Estimated Ages for Q Sub-Lineages in Mexico, Andes, Mongolia and Far East Asia and, in Comparative Populations.

	Country/Area/Population							
Lineage	Age ± SE (kya) (*N* a)							
	Mexico	Andes^b^	Mongolia	Far East Asiac,d	Altaiansd,e	Athapaskan(Na-Denè)f,g	Eskimo-Aleutsf,g	Indigenous communities-Andesd,h
Q-Total	21.1±4.4 (*178*)	23.4±3.9 (*196*)	14.5±2.9 (*19*)^i^	22.4±8.5 (*20*)	10.3±3.1 (*45*)			
Q1a-MEH2*				3.5±2.3 *(9)*				
Q1a3a-L53*					3.3±1.7 (*25*)			
Q1a3a1-L54* (xMexican cluster)	23.4±5.5 (*49*)^j^ 19.3±3.3 (*35*)	17.7±2.5 (*19*)^k^				5.6±2.3 (*5*)^l^		
Q1a3a1a-M3* (xAndean cluster)	19.3±4.5 (*121*)	23.7±4.4 (*161*)^m^ 20.5±4.3 (*131*)				3.4±1.6 (*21*)	7.4±3.4 (*6*)	
Q1a3a1b-L191	2.3±1.0 (*8*)							
Q1a3a1c-L330			6.5±2.5 (*14*)		2.9±1.2 (*20*)			
Q1a3a1a4-SA01		7.2±2.6 (*5*)^n^						5.9±2.1 (*16*)
Q1a3a1a5-M557		2.6±1.4 (*7*)						

a samples with duplicated alleles and partial repeats were excluded.

b Andes: Bolivia, Chile, Colombia, Ecuador, Peru.

c Koryaks (present study), Evens [59], Chukchi [78].

d DYS388 and DYS461 were not genotyped.

e Altai Republic [51].

f North America [49].

g DYS461 was not genotyped.

h Bolivia and Peru [56].

i Q-M120 (3) and Q-M25 (2) samples included, Q-L53* sample (1) not included because not genotyped for DYS461.

j 23.3±5.4 (48) without PV4.

k 20.7±3.7 (20) including outlier sample carrying DYS391 = 6 repeat.

l including one Mi'kmaq from Nova Scotia and one First Nation from British Columbia.

m with or without PV2.

n 15.4±6.5 (*6*) including outlier sample carrying DYS390 = 24 repeat; 18.5±7.7 (*6*) without DYS388 and DYS461; 9.1±3.0 (*5*) excluding outlier sample carrying DYS390 = 24 repeat and without DYS388 and DYS461.

The network of Q1a3a1a-M3* Y chromosomes includes North, Central and South American Y-chromosomes in a star-like shape, in agreement with an early entrance into America and wide expansion of this lineage in the entire continent. The central haplotype, which includes two Far Eastern Asian samples (one Koryak and one Even [59]), is most represented by Mexican and Andean subjects while Canadian samples are mainly observed as one and two steps derivative haplotypes. Regional expansions are present in all areas but are particular evident in the Andean and Canadian regions.

The network of the Q-L54* Y chromosomes is characterized by multiple reticulations, which suggest that they are part of different sub-clades still to be discovered. In spite the low frequency of these Y chromosomes, their preferential Mesoamerican distribution emerges. None of the L54* Y chromosomes is observed in Far Eastern Asian populations living along the entry route to the Americas; its sub-clade L330 underwent expansion in Mongolia and Southern-Altai, whereas the upstream intermediate L53* characterizes Northern-Altai (Figure 3), with a single observation in Mongolia.

## Discussion

Although numerous archaeological, linguistic and genetic studies have been performed to date, many aspects of the peopling of the Americas are still largely unresolved. Due to the extensive non-Native American admixture, which began with the arrival of Europeans after 1492, the main contribution to the reconstruction of Native American genetic origin and history has been provided by uniparental systems, which are not affected by the reshuffling effect of recombination. Unfortunately, the high historical rates of non-native male-mediated admixture into Native American communities have slowed down the investigation from the Y-chromosome perspective, which is essential for a complete picture of population movements and demographic events. This paper, in order to address this deficiency, investigates the genetic history of Central and South America using Y-chromosome variation and focusing exclusively on Y-chromosome haplogroup Q, which characterizes the great majority of modern Native Americans and is virtually the only native haplogroup in South America.

Two main founding lineages, Q1a3a1a-M3 and Q1a3a1-L54(xM3), have been observed in this study. Their variation in the Americas is not as restricted as was hitherto thought, giving genetic support to the Pleistocene/Holocene cultural variability in South America recently proposed by Neves and collaborators [60]. They display overlapping general distributions, but their diffusion patterns along the continent suggest they underwent different demographic processes. The most frequent, Q1a3a1a-M3*, shows a network which encompasses North, Central and South American chromosomes in a star-like shape indicative of a first general north-to-south diffusion followed by local expansions in Canada, Mexico and the Andean regions in agreement with a general north-to-south decreasing variance (*V*) and haplotype diversity (*h*) (Table S3). On the other hand, the network of Q1a3a1a1-L54* is mainly represented by single observations of well differentiated haplotypes and is characterized by many reticulations, indicative of the coexistence of deeper-rooted sub-lineages, with signs of expansions only in Mexico. The presence of these “expanded” haplotypes can explain the lower *h* encountered in Mexico (0.9344) in comparison with that observed in the Andean region (0.9883) in spite of the higher *V* (0.518 *vs* 0.451).

It is worth mentioning that, in these regions, important changes in environmental conditions were brought about by the development of different agricultural/animal domestication practices. In particular, while the subsistence economy of pre-Colombian Mexican society was strictly based on maize cultivation, in the Andes it was more varied, due to the presence of many different habitats and, unlike in Mexico, also took advantage of animal domestication. The consequent creation of ecological niches under different selection pressures may have determined changes in allele/haplotype frequency, as documented by the results of different studies on Central and South American populations [61]–[63]. In this scenario, even though Y-chromosome haplogroups may not be directly involved in activating this process, their frequency and variation could be influenced by genetic drift in areas where these evolutionary events took place. Accordingly, the different genetic structures of the Mexican and Andean areas, which emerge from the network analysis, are confirmed by the geographic distributions of their sub-clades, which reveal two distinct centers of evolution (Table 1), those rooted in the deeper L54 branch being associated with the Mexican population only, and those rooted in the M3 branch associated exclusively with the Peruvian (Andean) population.

With the exception of only one Q1a3-M346*(xL53) North American sample, all the Native American Q Y chromosomes sub-classified to date (N = 500) for L54 belong to this branch ([49] present paper). Therefore, Q-L54(xM3) should represent (at least in the southern part of the American continent) virtually all the samples previously classified as Q1a3-M346*/Q-M242*, and thus, be classified as the second most important Y-chromosome pan-American founder lineage. The frequencies observed in our population samples are in agreement with a distribution mainly along the northwest border of South America [20], [64], [65], as proposed in Bailliet et al. [66]. Of the L54 sub-clades, Q1a3a1b-L191 seems generally confined to the Western American coast. Our results reveal its presence in Mexico (1.6%) and Peru (0.3%) and, interestingly, its expansion time (3.6±1.6 kya) overlaps in Mexico (2.3±1.0 kya), coinciding with the historical timeframe of the Mayan Empire. Q1a3a1f-PV3 and Q1a3a1g-PV4 were identified in single Mexican subjects. However, Q1a3a1f-PV3 was found in association with a micro-variant (13.2 partial repeat) at the DYS19 *locus*, which on the YHRD database (http://www.yhrd.org – release 41 built on December 20, 2012 performed on 105,494 haplotypes within 782 populations) characterizes five subjects, three of them found in the Guatemalan-Mayan population [67], and two in Hispanic American admixed populations from United States [68]. The estimated age for chromosomes carrying that “partial repeat” allele dates back to the Pleistocene/Holocene boundary (Table 2) and, even though characterized by a large standard error, indicates an additional Mesoamerican differentiation of the most diffused L54. A further potential L54 branch could be represented by the peculiar DYS391–6 repeat allele, which characterizes two subjects from Panama and Colombia of the present study and seven Q(xM3) samples from Central America, five from Panama [69] and two from Nicaragua [70]. The absence in the large dataset of South American Y chromosomes carrying this microsatellite variant would suggest that this lineage was not involved in southern migrations. However, although in the past rare/partial repeat alleles within Y-STR *loci* were useful for assessing regional Y-chromosome variation or historical migrations and/or refining the resolution of the Y-chromosome tree [71]–[74], the phylogeography of these identified lineages must be confirmed by surveying a larger set of Native American samples.

The most diffused Q1a3a1a-M3 lineage appears to be more highly differentiated in the Andean region, where new sub-lineages were observed (Q1a3a1a4-SA01, Q1a3a1a5-M557, Q1a3a1a6-PV2), two at polymorphic frequencies. The SA01 marker was observed in Peru and Bolivia ([56], present paper) with an estimated variation age (7.2±2.6 kya, Table 3) compatible with the earliest appearance of agriculture in the Andean region. On the other hand, demographic events promoted by the diffusion of agriculture could explain the local differentiation and expansion of the Q-M3 Andean haplotype cluster, revealed by PCoA (Figure 2), whose estimated age has been evaluated at 4.0±1.2 kya (data not shown) and which includes the younger Q1a3a1a5-M557 sub-clade (2.6±1.4 kya, Table 3). In this context, it is worthwhile noting that the Y-chromosome haplotypes observed in eleven pre-Colombian male individuals buried in four different graves of the Peruvian archaeological site Tompullo 2 [75] overlap the modern haplotypes of the above mentioned M3 Andean cluster (Figure 2), thus providing evidence of a genetic continuity between extant Peruvian and pre-Colombian inhabitants. Taking into account that the Tompullo 2 site likely represents a short-lived settlement of Inca origins that existed during the Late Horizon (15^th^–16^th^ century) within the context of the *mitmaq* system (the colonization procedure adopted by the Incas to exploit new resources in new territories, based on movements of small population groups), the above mentioned Andean cluster, which is well separated from the bulk of the Q-M3 Andean Y chromosomes, could be the result of founder effects followed by an additional local expansion in a favorable ecological niche. Worth of note is the absence in our large Andean sample of the lineage Q1a3a1a1-M19 previously described in Native American groups from Colombia [20], [76] and recently observed in the Toba group from Argentina [77]. This observation indicates that the continental interior colonization of South America did not occur exclusively through the Pacific coastal route, but was followed by a trans-Andean crossing.

Regarding the Asian distribution of the two Native American Q founding lineages, Q-M3* has been found in different Far East Asian groups, whereas, at present, only a potential Q-L54* has been observed in one Chukchi from Northern Siberia [78]. However, since this L54 sample has not been classified for the L330 marker, which characterizes the Asian branch of Q-L54, and occupies a position close to the Mexican haplotypes (not shown) when included in the L54* network, at present any interpretation of this result (new Asian lineage, remnant of an ancestral state, trace of forward or back-migration) is premature. On the contrary, some information can be obtained by the analysis of Asian M3 Y chromosomes. The presence of Far East Asian samples in the central haplotype of the M3* network and of subjects from the Northwest territories of Canada in one- and two-step derivative haplotypes as well as the large haplotype sharing between Central and Southern America and the general north-to-south decreasing pattern of *V* and *h*, lead to the following considerations: i) haplogroup Q1a3a1a-M3 was probably the protagonist of a swift migration that originated a uniform pattern of distribution along the American continent; ii) the local differentiation identified in the Andean region due to long-term isolations of Q-M3 chromosomes supports an early arrival of this lineage in South America; iii) the northern territories of the Americas, despite subsequent arrival(s), retain the footprint of this first migration; and iv) even though M3 likely arose in Beringia, the extant North East Asian representatives could better represent the result of a back migration rather than be direct M3 Beringian descendants. With this regard, it is noteworthy that, after a Beringian population expansion concomitant with the entry to America, more recent (during the Holocene) contacts among Asian and American circum-arctic populations were proposed to explain the distribution of morphological characters [79]. The presence of M3 subjects in some Far East Asian groups could also be due to recent contacts (gene flows) with modern northern Native Americans. Their presence in populations such as coastal Chukchi [78], Evens [59] and Koryaks (present study), characterized by a subsistence economy which integrates land-based activities (reindeer herding or dog breeding) combined with marine animal hunting, gives support to this interpretation. Moreover, the presence in these North Eastern groups of chromosomes belonging to haplogroup Q1a-MEH2*, which characterizes the 4 ky-old Palaeo-Eskimo individual of Saqqaq culture (marine hunting), confirms the genetic continuity between modern and Palaeo-Eskimo populations. The estimated age of Q1a-MEH2* microsatellite variation obtained after having included our samples (Table 3) is in agreement with the scenario that the hypothesized crossing of the Bering Straits occurred 5.5 kya, independent from previous entry waves [39]. Accordingly, Q1a-MEH2, previously described in different indigenous groups from Far North-Eastern Russia [59], [78], [80] and Southeast Alaska [49], was not found in Central and South America.

Finally, our Mongolian samples (Table S5), which are from a geographic area lying over the Altai region of Mongolia (Western Mongolia) and the northern part of the country (including also the territory of Kazakh and Buryat ethnic groups, historically scattered between Balkhas and Baikal Lakes), share with southern Altaians and southern Siberian populations [51] the L54 sub-haplogroup Q1a3a1c-L330, and with northern Altaians the phylogenetically upstream Q-L53* observed in one subject (the only one from Eastern Mongolia) (Figure 3 and Table S5). These observations are in agreement with the hypothesis that the Native American ancestral population comes from somewhere in southern Siberia, between the Altai Mountains (which acted as a genetic and cultural wall) and the Lake Baikal region [16]. Thus, it is likely that the ancestral form of the L54 Y chromosomes was already comprised in the probable Asian source population(s), which, as supported by archaeological records [81], in prehistoric times and long before the peopling of the New Continent, moved eastwards during the Beringian standstill. In this setting, they evolved into the Native American Y-chromosome founding lineages (Q-L54* and Q-M3) [3], which began a rapid southwards diffusion following climatic ameliorations. In this context, it is noteworthy that the only Q sample from southeastern Europe was Ukrainian [82], which is now classified as M346*(xL53, L54, M3, L191, L330, L401, L323), suggesting that the spread of haplogroup Q westwards probably occurred via Central Asia prior to the occurrence of the L53 and L54 mutations.

In this complex scenario, our data (1) are in agreement with the southern Siberia origin of Native American ancestral populations and the differentiation of both Native American Q founding lineages in Beringia; and (2) support a concomitant arrival of the Q-M3 and Q-L54* founding lineages in Mesoamerica (Table 2 and 3) and, therefore, the model proposed by Sandoval et al. [65] where Mexico was the recipient of the first wave of migration. However, our data also (3) evidence different patterns of evolution for the two founding lineages in the Mexican plateau and the Andean area, which can be explained by local differentiations of the two founder lineages after their rapid arrival first in Mexico and then, along the Pacific coast, in the Andean region. The differentiations observed in Mexico and the Andean region can be mainly ascribable to demographic expansions triggered by the introduction of agriculture and the flourishing of the Great Empires.

## Materials and Methods

### The sample

We analyzed 463 Y chromosomes belonging to haplogroup Q mainly from the Sorenson Molecular Genealogy Foundation (SMGF – http://www.smgf.org) database (Table 1). Candidates for haplogroup Q Y chromosomes were identified and selected according to genealogical, geographic and STR haplotype information. As for Native Americans, in the initial SMGF sample of 15,000 DNA donors from 31 countries ranging from North to South America, we identified 4,037 potential Native American subjects with paternal ancestors' birthplaces in the Americas for at least three generations. Among these, 2,025 presented STR haplotypes associated with haplogroup Q, according to the prediction algorithms. As for Asians, the initial sample included 1,179 unrelated males: 1,145 subjects from different areas of Mongolia and 34 Koryaks from the Kamchatka Peninsula.

The haplogroup prediction process was carried out using the SMGF prediction algorithm drawn on data from a subset of samples genotyped for 37 STR *loci* and signature haplogroup markers. A second publicly available Y-chromosome haplogroup predictor algorithm [83] was used for validation purposes. Confidence scores were also evaluated.

### Ethics Statement

All experimental procedures and individual written informed consent, obtained from all donors, were reviewed and approved by the Western Institutional Review Board, Olympia, Washington (USA) and by the Ethics Committee for Clinical Experimentation of the University of Pavia, Board minutes of the 5th of October 2010.

### STR and SNP genotyping

All Native American and Mongolian samples were typed for 37 Y-STRs (four of which, YCAIIa/b, DYS385a/b, DYS459, DYS464, are duplicated *loci*) according to the SMGF website.

All samples predicted to be K-M9 and Q-M3 were analyzed for the haplogroup Q signature marker M242 [84] and all the M242-positive samples were hierarchically analyzed for 22 downstream markers by RFLP and/or DHPLC and/or Sanger sequencing of the pertinent PCR fragment. Primers and methods used for each marker are provided in Table S1.

Haplogroup nomenclature is according to Dulik et al. [49], [51] and updated in agreement with the YCC [85] rules (http://ycc.biosci.arizona.edu).

### Statistical and phylogenetic analyses

Haplogroup frequencies were calculated by direct counting. Diversity parameters (number of shared haplotypes, haplotype diversity) were calculated only using the minimal Haplotype (minHt – DYS19, DYS389I, DYS389B, DYS390, DYS391, DYS392, DYS393, DYS385a/b – [86], [87] disregarding the duplicated DYS385a/b *locus*. All these analyses were performed using the Arlequin v. 3.5 software [88]. Samples with atypical alleles (duplicated and micro-variant alleles) were not considered in the evaluation of diversity parameters.

Principal Coordinates Analysis (PCoA), on pairwise, individual-by-individual genetic distances, based on thirty-three *loci* Y-STR haplotypes, was performed using Excel software implemented by GenAlEx 6.4 software [89]. The YCAIIa/b, DYS385a/b, DYS459 and DYS464 *loci* were not included in the analyses because of the impossibility of assigning each allele to one or the other *locus* of these systems. One sample with duplicated DYS390 *locus* was also excluded from this analysis.

Phylogenetic relationships among haplogroup Q Y chromosomes were investigated through Median-Joining (MJ) networks [90] generated with Network 4.6.1 and Network Publisher 2.0 software (http://www.fluxus-engineering.com), after having processed data with the reduced-median method [91]. Networks were generated only using the minHt, by weighting each *locus* proportionally to the inverse of its repeat variance and disregarding the duplicated DYS385a/b *locus*.

The age of microsatellite variation (coalescent time or expansion time) within haplogroup Q and its sub-lineages was evaluated by the methodology of Zhivotovsky et al. [57] as modified according to Sengupta et al. [52]. In particular, it was estimated as the average squared difference in the number of repeats between all current chromosomes and the median haplotype, averaged over microsatellite loci and divided by the effective mutation rate of 6.9×10 per 25 years, with the SE computed over loci (details, in Appendix A of Sengupta et al. [52]). A microsatellite evolutionary effective mutation rate of 6.9×10^−4^ per generation (25 years) was used [52], since it is suitable for time depths, such as those explored in this study, where the elapsed time frame is ≥1000 years or ∼40 generations [58].

Although caution should be used in the dating procedures because of the lack of a reliable mutation rate for Y-chromosome STR *loci* – also the use of pedigree-based mutation rate gave archaeologically inconsistent results [51] – estimated ages give us an idea concerning the temporal relationships existing among the different sub-lineages within the haplogroup.

## Supporting Information

Table S1 **Description of analyzed markers.**
(XLSX)

Table S2 **Thirty-three** ***loci*** **Y-STR haplotypes in Native American Q chromosomes.**
(XLSX)

Table S3 **Variance and haplotype diversity values for haplogroups L54 and M3.**
(XLSX)

Table S4 **Seven loci Y-STR haplotypes in the analyzed samples.**
(XLSX)

Table S5 **Mongolian sample description and haplogroup classification.**
(XLSX)
